# Goal-Focused Emotion-Regulation Therapy (GET) for young adult survivors of testicular cancer: a pilot randomized controlled trial of a biobehavioral intervention protocol

**DOI:** 10.1186/s13063-020-04242-0

**Published:** 2020-04-14

**Authors:** Michael A. Hoyt, Ashley Wei-Ting Wang, Sean J. Ryan, Elizabeth C. Breen, Jennifer S. Cheavens, Christian J. Nelson

**Affiliations:** 1 Department of Population Health and Disease Prevention and the Chao Family Comprehensive Cancer Center, University of California, Irvine, 653 E Peltason Drive, Irvine, CA 95697-3957 USA; 2 Department of Psychology, Soochow University, Shinlin, Taiwan; 3 Department of Psychology, Graduate Center, City University of New York, New York, NY USA; 4 Cousins Center for Psychoneuroimmunology, University of California, Los Angeles, Los Angeles, CA USA; 5 Department of Psychology, Ohio State University, Columbus, OH USA; 6 Department of Psychiatry and Behavioral Sciences, Memorial Sloan Kettering Cancer Center, New York, NY USA

**Keywords:** Biobehavioral, Intervention, Testicular cancer, Goal, Young adult, Cancer survivorship

## Abstract

**Background:**

Testicular cancer diagnosis and treatment, especially given its threat to sexuality and reproductive health, can be distressing in the formative period of young adulthood and the majority of young survivors experience impairing, distressing, and modifiable adverse outcomes that can persist long after medical treatment. These include psychological distress, impairment in pursuit of life goals, persistent physical side effects, elevated risk of secondary malignancies and chronic illness, and biobehavioral burden (e.g., enhanced inflammation, dysregulated diurnal stress hormones). However, few targeted interventions exist to assist young survivors in renegotiating life goals and regulating cancer-related emotions, and none focus on reducing the burden of morbidity via biobehavioral mechanisms. This paper describes the methodology of a randomized controlled biobehavioral trial designed to investigate the feasibility and preliminary impact of a novel intervention, Goal-focused Emotion-Regulation Therapy (GET), aimed at improving distress symptoms, emotion regulation, goal navigation skills, and stress-sensitive biomarkers in young adult testicular cancer patients.

**Methods:**

Participants will be randomized to receive six sessions of GET or Individual Supportive Therapy (ISP) delivered over 8 weeks. In addition to indicators of intervention feasibility, we will measure primary (depressive and anxiety symptoms) and secondary (emotion regulation and goal navigation skills, career confusion) psychological outcomes prior to (T_0_), immediately after (T_1_), and 12 weeks after (T_2_) intervention. Additionally, identified biomarkers will be measured at baseline and at T_2_.

**Discussion:**

GET may have the potential to improve self-regulation across biobehavioral domains, improve overall cancer adjustment, and address the need for targeted supportive care interventions for young adult cancer survivors.

**Trial registration:**

Clinicaltrials.gov, NCT04150848. Registered on 28 October 2019.

## Background

The development of effective interventions that prevent, control, and eliminate physical, psychological, and behavioral adverse effects of cancer in young adult survivors is a public health priority [1]. Testicular cancer is the most prevalent non-skin cancer among men in late adolescence and early adulthood, and rates of new diagnoses have been rising over the last decade [2]. Ensuring robust health-related quality of life is essential in this group, as they face both psychological and physical impact from potential loss of a reproductive organ and long-term functional impacts of chemotherapy, radiation therapy, and/or surgery [3–5]. Further, long-term sequelae are more severe and persistent in those receiving chemotherapy, and include peripheral neuropathy, hearing loss, hypogonadism, infertility, secondary malignancies, long-term hearing loss, sexual dysfunction, and development of cardiovascular disease [4, 6–10].

Psychosocial impact is also substantial as the prevalence of depressive symptoms and anxiety in testicular cancer exceeds that in the general population [3]. Common concerns include body image disruption, social relationships, fertility and sexual distress, masculinity threat, work-related problems, and worry about the future [5, 11–13] and nearly two-thirds of testicular cancer survivors report unmet survivorship needs [13–15], most commonly relating to supportive care, survivorship information, managing distress, fertility, relationships and self-image, and occupational problems [14–17]. Yet there is a paucity of psychosocial supportive cancer interventions for young adults. In a systematic review, Walker et al. [18] identified only 18 psychosocial interventions for young adult survivors, and only 8 of these were tested in a randomized controlled trial. Also, none of these interventions were tailored to the needs of young men, and none targeted developmentally informed processes of self-regulation.

Adjustment to challenged goals constitutes adaptive self-regulation [19] and may be particularly critical when cancer occurs in early adulthood [20, 21]. Cancer diagnosis and treatment present circumstances that challenge the achievement and pursuit of meaningful life and developmentally timed goals (e.g., pursuit and maintenance of dating and sexual relationships, identification of values-driven occupational pursuits, achievement of independence from parents) [22, 23]. Cancer-related goal disturbances are associated with chemotherapy receipt and a host of behavioral and psychological symptoms, including depression, fatigue, pain, and cognitive complaints [24]. Concerns about the achievement of life goals are especially distressing for adolescents and young adult survivors [25–27], who are negotiating greater autonomy across life domains and are oriented toward achievement of future goals. Goal navigation skills include the ability to identify new and existing goals; together with emotion-regulating coping behaviors, they have the potential to support cancer-related well-being [28–31], and they might play a role in regulation of stress-sensitive biobehavioral factors, including inflammatory and neuroendocrine processes [32, 33].

This manuscript describes the methodology of a randomized controlled biobehavioral pilot trial designed to investigate the feasibility of Goal-focused Emotion-Regulation Therapy (GET), a novel intervention aimed at improving distress symptoms, self-regulation (i.e., emotion regulation and goal navigation skills), and stress-sensitive biomarkers in young adult testicular cancer patients with the overarching goal of establishing whether a future larger-scale trial can and should be conducted in this manner. The intervention will be administered in six sessions delivered over 8 weeks to a sample of 60 young adult (ages 18–39 years) recent testicular cancer survivors. As an external pilot study, a major focus will be on testing data collection procedures and measurement strategies, optimizing the recruitment plan, testing randomization, and informing appropriate sample size estimates. Examination of the integrity and acceptability of the study protocol will be of primary focus.

### Hypotheses

We hypothesize that young adult testicular cancer survivors will confirm the formative findings that the GET intervention is feasible, tolerable, and acceptable. Furthermore, we expect that, in comparison to those in Individual Supportive Therapy (ISP), participants receiving GET will have a measurable, positive improvement on potential primary and secondary outcomes. Primary outcomes of consideration are change in psychological distress (i.e., depressive and anxiety symptoms) from baseline to 12 weeks post intervention and change in systemic inflammation markers (i.e., IL-6, IL-1ra, CRP, sTNFαRII) and salivary cortisol regulation (i.e., diurnal cortisol slope and daily cortisol output) from baseline to post intervention. However, results related to changes in distress symptoms will inform sample size estimates in future trials. Of additional focus are changes in self-regulatory processes (i.e., emotion regulation and goal navigation skills, career confusion) from baseline to 12 weeks post intervention.

## Methods

### Study design

**Fig. 1:**
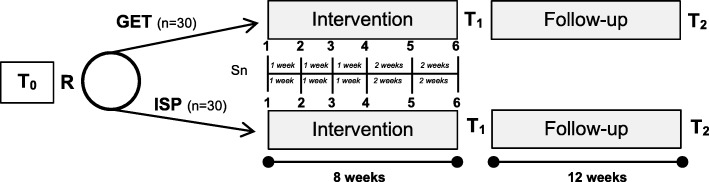
Study schematic. GET Goal-Focused Emotion-Regulation Therapy, ISP Individual Supportive Psychotherapy, R randomization, Sn session number, T_0_ baseline, T_1_ post Intervention, T_2_ 12-week follow-up

Study goals and design were informed in part by the SPIRIT guidelines [34], previous cancer control intervention research [35, 36], and models of translational research for behavioral interventions [37]. This study will utilize a randomized, controlled, repeated-measures design to investigate the feasibility of studying GET relative to a well-matched comparator (i.e., ISP) to improve identified variables in young adult testicular cancer patients. See Fig. 1.

The replication and extension of the promising and preliminary results of our formative research (described in the following) will contribute to satisfying the critical need for accessible, feasible, effective, and scalable interventions for young adult survivors with the goal of ameliorating long-term negative effects of cancer and cancer treatment. Participants will be 60 young adults with testicular cancer who have completed treatment (including chemotherapy) and will be randomized 1:1 into GET or ISP using a block of 10 randomization design. A central randomization scheme will be used in which a research recruiter will contact a central methods center by secure computer for allocation assignment for newly enrolled participants to maintain concealment of the randomization sequence. The sequence will be generated by a computer randomization program.

Sample size was determined by balancing realistic recruitment estimates and minimal sample requirements for planned analyses. Informed consent will be obtained individually following eligibility screening and prior to data collection. The interventions will be delivered face to face in a clinical setting. However, to enhance accessibility, telephone sessions will also be possible for those with accessibility barriers.

Participants will complete questionnaire assessments at baseline (T_0_), at the completion of the last GET or ISP session (T_1_), and at 12 weeks following completion of the last GET or ISP session (T_2_). In addition, biological specimens will be collected at T_0_ and at T_1_. These include the collection of plasma via venipuncture (in the clinic) and samples of saliva (at home). Saliva is collected over 2 days at each assessment point upon awakening, 30 min later, 8 h later, and at bedtime [38]. Questionnaire data will be collected via an online password-protected data collection platform. All participants will be offered $50 at each data collection point for completion of assessments and collection of biomarkers (and $25 without biomarker assessment).

All study procedures will be reviewed and approved by the University of California, Irvine and Memorial Sloan Kettering Cancer Center Institutional Review Boards.

Given the assessed low risk of trial participation, serious adverse events and harms are not anticipated; no formal stopping rules are designated. Likewise, a study-specific data monitoring committee was not considered. However, the institutional data safety board for cancer studies is available for consultation and the investigational team will meet weekly to monitor study progress and activities.

### Participants

Participants will be young adults with a diagnosis of testicular cancer who have completed active treatment (surgery + chemotherapy) and present with elevated distress and/or deficits in goal navigation skills. We will enroll participants from the Chao Family Comprehensive Cancer at the University of California, Irvine and Memorial Sloan Kettering Cancer Center, and various young adult community-based support groups or children’s hospital settings.

### Eligibility

Participants will be initially identified through electronic medical record screening or identified by clinic personnel, or will self-identify in response to study announcements.

Determination for study eligibility will occur via screening by a research study assistant. Participants eligible for this study will be those who: are between the ages of 18 and 39 years at the time of consent; have a confirmed diagnosis of testis cancer (any stage); have completed chemotherapy for testis cancer within 2 years prior to consent; have fluency in English (per self-report); and exhibit suboptimal self-regulation, evidenced by a score of 1.8 or below on the goal navigation scale [28] or a score of 4 or greater on the Distress Thermometer (DT) [39].

Exclusion criteria include: lifetime history of bipolar disorder, schizophrenia, or schizoaffective disorder (per self-report); an active suicide plan; presence of a disorder that compromises comprehension of assessments or informed consent information (e.g., dementia); and self-reported medical condition or medication use known to confound measures of systemic inflammation (e.g., autoimmune disorder, active infection; myocardial infarction or stroke in the last 6 months; recent vaccination for viral disease); and daily smoking [38, 40].

Participation will not require alteration to usual care including use of any medication or psychotherapy.

### Intervention conditions and delivery

#### Goal-Focused Emotion-Regulation Therapy

##### Development of GET intervention

The identification of core components and the development of the GET intervention come from preliminary empirical work with young adult testicular cancer survivors. First, an in-depth interview study of a diverse sample (*N* = 21; 48% ethnic minority) of young adult survivors of testicular cancer [41] revealed that young men perceive that life goals are an important aspect of adapting to testicular cancer, and identified goal and values clarification, engagement and disengagement from life goals, and the ability to express and process emotional responses to disrupted goals among critical self-regulation skills regarding behavioral and emotional responses to cancer-related stressors. Second, they identified a preference for action orientation, positivity, achievement, and practical realism. This included an inclination toward the use of an active, approach-oriented stance to pursuing prior goals and/or reengaging with new goals after cancer. Third, findings suggested that when goal navigation capacity was low or impaired, young men were less likely to benefit from their efforts at approach-oriented coping.

In a follow-up observational study of the same population (*N* = 171), Hoyt et al. [28] identified measurable goal-related processes including “goal navigation”, or the capacity to identify and pursue valued life goals. Goal navigation demonstrated cross-sectional associations with aspects of cancer-related adjustment. In regression analyses controlling for age and time since diagnosis, goal navigation was positively associated with emotional (*B* = 0.35, *p* < 0.001), social (*B* = 0.24, *p* < 0.01), and functional (*B* = 0.28, *p* < 0.001) well-being [41]. Further, a theoretical model of goal navigation using a self-regulation framework was identified and tested [42]. Goal navigation skills were negatively correlated with depressive symptoms (*r* = − 0.41, *p* < 0.01) and positively correlated with physical functioning (*r* = 0.28, *p* < 0.01), and these were mediated by possessing a sense of meaning in goal pursuit (indirect effects: depressive symptoms (*r* = − 0.50, *p* < 0.05), physical health (*r* = 0.34, *p* < 0.05)) and emotion regulation skill (indirect effects: depressive symptoms (*r* = − 0.08, *p* < 0.05), physical health (*r* = 0.11, *p* < 0.05)).

In a follow-up, at-home monitoring study (unpublished data) to examine associations of goal navigation skill, emotion regulation, and salivary markers of diurnal cortisol and α-amylase, the use of emotion regulation skills and salivary measures were collected daily for 2 days. Low levels of goal navigation capacity were significantly associated with greater use of less constructive emotion regulation strategies and higher daily salivary cortisol output (area under the curve). Poor self-regulation skills underscore dysregulation of biological processes with strong potential to lead to declines in physical health and mood.

Taken together, these findings underscored development of GET and an initial intervention manual which was tested to establish the feasibility and acceptability for the patient population. This included initial patient focus group interviews, as well as a series of individual patient interviews to gain feedback into session content, intervention length and format, and structured at-home components. A revised manual emerged that, based on findings, included more “normalization of experience”, opportunities to address cancer-specific topics (e.g., cancer-related disclosure, self-care after cancer), and bolstering of emotion regulation skill-building.

Next, the revised intervention was pilot tested with six young adult testicular cancer patients to assess tolerability, acceptability, adherence, and retention. This was done through examination of treatment completion and engagement with at-home exercises as well as in-depth post-participation interviews. Supporting tolerability and acceptability, all six participants completed all study sessions with no adverse events. Also, participants’ engagement with assigned at-home exercises were coded as “attempted/not attempted” to inform adherence. Moderate adherence was observed with 78% of assigned activities attempted. Interview transcripts were analyzed with a targeted inductive procedure of thematic analysis. This involved thorough reading and review of transcripts, synthesis of key conceptual findings, and the generation of descriptive conclusions. Conclusions identified eagerness for a survivorship intervention, affirmation of the importance of the identified intervention targets, satisfaction with the intervention length and depth, and desire for study interventionists to challenge participants to “go deeper” in the process of identifying and processing emotions. Participants also identified the desire to hear more perspectives from other testicular cancer survivors. Finally, the manual was further refined based on interview findings. This included the inclusion of patient quotes in study participant hand-outs.

##### GET intervention components

GET is a six-session intervention delivered over 8 weeks to enhance self-regulation through improved goal navigation skills, improved sense of meaning and purpose, and better ability to regulate specific emotional responses. GET draws heavily from the principles of Hope Therapy [43], with an emphasis on goal navigation skill-building. Components of Hope Therapy have been used successfully in cancer survivorship interventions [44]. This includes work on goal-setting with a focus on assessing progress toward achieving specific, realistic, and measurable goals. Patients identify value-derived goals (i.e., goals for the most important domains of one’s life) and ones sufficiently important to sustain movement toward them in the short-term future. They discuss their goal possibilities, providing a forum to ensure that goals are manageable and consistent with identified values. Patients learn strategies to refine their goals (e.g., approaching goals rather than avoiding obstacles, defining markers of progress), generate pathways to goals, and address potential obstacles and blockages. Additionally, goals provided the context for demonstrations of agentic thinking (e.g., I will be able to do this) and interventions to increase agentic thinking. Specific attention is given to career/education-related goals. Emotion regulation components include basic cognitive restructuring skills, cognitive distancing, and coping efficacy skills (matching the correct coping skill to specific circumstances) (see Table 1).

**Table 1:** GET session guide

Session	Session focus
1	Review of cancer-related experiences and influences on goal pursuits; psychoeducation regarding emotions, skills, and values
2	Values clarifications and emotional awareness
3	Achievability of goals, cognitive skills training
4	Goal pathway mapping, navigating blocked goals and re-directing energy
5	Goal motivation and agentic actions, self-care behavior
6	Goal pursuits moving forward

*GET* Goal-Focused Emotion-Regulation Therapy

#### Individual Supportive Psychotherapy

ISP will be utilized as the comparison treatment condition in this study. ISP is one of the predominant approaches to community-based supportive care in psychosocial oncology. In this study, ISP is adapted from the Supportive Group Psychotherapy manualized intervention [45], and subsequently adapted further for use in our pilot work for young adult testicular cancer patients. This intervention includes six sessions of Individual Supportive Psychotherapy utilizing an approach based on models described by Rogers [46, 47] and Block [48], including components of genuineness, unconditional positive regard, and empathic understanding through reassurance, explanation, guidance, suggestion, encouragement, affecting changes in patient’s environment, and permission for catharsis [48]. ISP also emphasizes maintaining focus on the cancer experience, supporting participants in the “here and now”, fostering expression of emotion and discussion of difficult topics, and creating a sense of being understood [45]. The manual offers instructions on how to avoid therapeutic techniques associated with other treatment modalities (e.g., CBT, interpersonal psychotherapy).

### Intervention delivery

GET and ISP will be delivered by at least a master’s-level mental health clinician interventionist who will receive intensive training prior to delivering the intervention and regular supervision after each session. All interventionists will be male. Therapists without prior GET experience will conduct one or two training cases with patients not enrolled in the proposed study. Participants consented as training cases will not be randomized and will not complete any formal assessments.

Sessions will be audio recorded so that treatment fidelity can be regularly monitored and independently rated by trained research assistants.

Participants will be permitted to continue to see any outside mental health professionals during the trial. In these instances, outside interventions will be documented and controlled for during data analysis.

### Data collection and measurement

#### Data collection

Data will be collected at each identified time point and during the eligibility screening process. A research study assistant, blinded to the study objectives and randomization, administers the screening questionnaire verbally. Baseline and T_1_ data collection will be completed during an in-person meeting where they complete questionnaires via computer and provide a blood sample. Saliva will be collected over 2 days at home in close proximity to these data collection points. Participants can complete T_2_ questionnaires remotely by computer. Following study completion, we will conduct a medical record review. Finally, a subset of GET-assigned participants (*n* = 6) will be asked to participate in a qualitative exit interview to gather subjective experiences of participation. See Table 2 for the data collection plan.

**Table 2:** Data collection scheme

	Enrollment	Assessment					Close-out
Timepoint	Pre T_0_	T_0_	Post baseline	T_1_	T_2_	Post T_2_	Chart review
Screening							
Eligibility screen	X						
Informed consent	X						
Allocation			X				
Interventions							
GET							
ISP							
Assessments							
Hospital Anxiety and Depression Scale (HADS)		X		X	X		
Goal Navigation (CAYA-T)		X		X	X		
Emotion Regulation Questionnaire (ERQ)		X		X	X		
Career Thoughts Inventory (CTI)		X		X	X		
Blood draw		X		X			
At-home saliva sampling		X		X			
Demographics		X					
Semi-structured interview						X	
Medical chart review							X

*CAYAQ-T* Cancer Assessment for Young Adults—Testicular, *GET* Goal-Focused Emotion-Regulation Therapy, *ISP* Individual Supportive Psychotherapy, *T*_*0*_ baseline, *T*_*1*_ post Intervention, *T*_*2*_ 12-week follow-up

#### Measures

##### Screening measures

Two measures are used in participant screening, as described earlier: the Distress Thermometer (DT) and the Cancer Assessment for Young Adults (CAYA-T; Goal Navigation Skill subscale). The DT [39] is a single-item visual analog scale used to screen cancer patients and ICs for psychological distress with a 0–10 range accompanied by a 34-item problem checklist [49]. Extensive research has identified a score of 4 or greater for identifying clinically significant distress [50, 51].

Goal navigation skill is measured by the goal navigation scale of the CAYA-T [28]. The CAYA-T has been validated on a sample of young adult men with testicular cancer and has demonstrated good psychometric properties in this population.

##### Depression and anxiety symptoms

Depression and anxiety will be assessed using the Hospital Anxiety and Depression Scale (HADS) [52], a 14-item self-rated questionnaire of overall psychological distress, well tested in cancer populations [53].

##### Emotion regulation

Emotion regulation skills will be measured by the Emotion Regulation Questionnaire (ERQ) [54]. The ERQ is a widely used 10-item scale designed to measure respondents’ tendency to regulate their emotions in two ways: cognitive reappraisal and expressive suppression. Respondents answer each item on a 7-point scale ranging from 1 (strongly disagree) to 7 (strongly agree).

##### Goal navigation

Goal navigation skill is measured by the goal navigation scale of the CAYA-T, as already described. In addition, goal regulation capacity will be measured by agency and pathways. The Hope Scale [55] is a 12-item measure of a respondent’s level of hope. In particular, the scale is divided into two subscales that comprise Snyder’s cognitive model of hope: agency (i.e., goal-directed energy) and pathways (i.e., planning to accomplish goals).

##### Career confusion

Career confusion will be measured with the Career Thoughts Inventory (CTI) [56]. The CTI is a 48-item measure that is designed to assess career navigation difficulty and is normed on adult populations. The CTI yields a total score as well as scores on three construct scales: decision-making confusion, commitment anxiety, and external conflict.

##### Demographic and clinical information

Demographic information, past/current psychosocial service use, support needs, intervention preferences, and perceived barriers are assessed through Likert-scale ratings and open-ended items. Clinical information will also be assessed via medical record review and via self-report. In addition, we will thoroughly assess for medical comorbidities and physical health symptoms.

#### Biological assessments

##### Inflammatory markers

We will focus on five inflammatory biomarkers that have proinflammatory function, are associated with distress symptoms, and/or have angiogenic properties: IL-6, IL-1RA, CRP, sTNFrR2, and VEGF. Blood samples for circulating inflammatory markers will be collected between the hours of 8 a.m. and 4 p.m. by a trained phlebotomist by venipuncture into EDTA heparinized tubes, placed on ice, centrifuged for acquisition of plasma within 30 min, and stored at − 80 °C for subsequent batch testing. Plasma levels of identified markers will be determined by enzyme-linked immunosorbent assay according to the assay manufacturer’s protocols. CRP will be determined by a high-sensitivity enzyme-linked immunosorbent assay according to the assay manufacturer’s protocol. All samples will be run in duplicate, and assays will be repeated on two separate days for sTNFr and IL-6; intraassay and interassay mean levels will be used in all analyses.

##### Diurnal salivary stress biomarkers

We will focus on two salivary stress biomarkers. These include *s*alivary cortisol, as a downstream marker of HPA activity, and salivary α-amylase (sAA), as a proxy measure of SAM activation; they can be measured concomitantly [57] and together offer a comprehensive view of physiological stress, as differing diagnostic and treatment effects have been observed between these markers [58]. Both salivary cortisol and sAA follow a distinct diurnal rhythm: cortisol levels peak approximately 30 min after awakening and decrease throughout the day, whereas sAA has a pronounced decline after awakening followed by an increase across the day [57].

Diurnal rhythm in salivary markers will be measured over 2 days at baseline and post intervention. Participants will collect saliva samples in their natural environment upon awakening, 30 min later, 8 h later, and at bedtime as recommended by Nicolson [38]. Participants will be instructed to go about their normal daily activities on the days of data collection and will complete a diary to assess relevant health behaviors (e.g., caffeine use). To avoid sample contamination, they will be instructed to avoid brushing their teeth, eating, or drinking within 20 min before sampling. Participants will be instructed to keep samples refrigerated prior to returning them to the research laboratory and returned salivettes will be stored in a freezer at – 80 °C until analyzed. After data collection is complete, salivary markers will be analyzed with a time-resolved fluorescence immunoassay. Several indices will be computed including diurnal slope, area under the daily curve, cortisol awakening response, and total daily cortisol output.

#### Evaluation data

##### Qualitative interview

Six participants who complete the GET sessions will be invited to complete a semi-structured qualitative interview, using a semi-structured interview guide. Consecutive participants completing GET will be offered participation in the interviews until six interviews are completed. The interview will focus on understanding feasibility, tolerability, and acceptability of GET and overall trial participation from the patient’s perspective. Through the interviews, participants will provide information about their experience of participating in GET, including challenges to their participation and elements that they found most and least appealing. Qualitative methods will be employed to elicit and evaluate participant responses to the semi-structured interview. The interview transcripts will be analyzed with a targeted inductive procedure of qualitative thematic text analysis [59]. This process will involve thorough reading and review of transcripts by an analysis team; synthesizing key conceptual findings of each transcript; identifying key conceptual findings across all transcripts; and generating descriptive and interpretive themes for the entire data set.

##### Patient satisfaction

A 16-item questionnaire administered after the last session was adapted from a measure used in currently/past federally funded intervention studies. Items query subjects’ responses to the various components of the intervention (e.g., content, timing, and length of sessions), and will be used for intervention modification.

#### Descriptive data

We will use descriptive statistics to summarize multiple aspects of study feasibility, including acceptability (percentage of approached and eligible men who consent to the study), tolerability (percentage of consented and enrolled men who complete the study in both arms), and adherence (descriptive statistics summarizing the rate of patients completing the scheduled sessions for both arms).

#### Data analysis

Standard descriptive statistics will be used to report baseline participant characteristics by condition. For continuous variables with markedly non-normal or skewed distributions, appropriate transformations may be required, such as natural logarithms, and will be applied as necessary and appropriate. We will perform analysis according to the intention-to-treat principle (i.e., participants will be analyzed according to the treatment group to which they will be randomly allocated regardless of dropout or treatment adherence status).

Change from baseline will be calculated across variables. Follow-up scores on the identified variables will be compared between study conditions (adjusting for baseline), via repeated-measures analysis of covariance (ANCOVA). We will also reference established cutoff values for clinically meaningful differences for scores on the HADS to identify ranges and patterns of change. Mixed-effects models will be used to model the scores over time for each group. Time since diagnosis, education, marital status, medical comorbidities, and age will be examined as potential covariates to model.

## Discussion

This trial will evaluate the feasibility of an individual biobehavioral intervention targeted to the needs of young adult men treated for testicular cancer. There is significant need for *appropriate* behavioral intervention in order to decrease the psychological, physical, and social toll of diagnosis and treatment. Meeting these needs is challenging, as men (particularly younger men) tend not to seek professional help for distress [60]. In fact, there is increasing evidence that men are reluctant to seek professional help due to “traditional” masculine attitudes [61], highlighting the need to develop interventions that are both accessible and acceptable to men. A meta-synthesis of qualitative studies [62] investigating the accessibility and acceptability of self-management support interventions (both online and offline) for men with long-term conditions, including cancer, found that self-regulation interventions may be particularly more acceptable to men, as they enable the men to take control over managing their distress (promoting self-sufficiency and independence).

A central target of the developed intervention is goal navigation skills. These include the ability to identify new and existing goals, which serves to support cancer-related well-being [28]. A second targeted self-regulatory process is emotion regulation. Indeed, difficulty regulating emotions is common across psychological disorders [63]. Emotion regulation involves generating emotional responses, as well as modulating the manner in which one alters, experiences, and communicates such responses [64].

Specific limitations to this study should be acknowledged. It is possible that elements of GET could be accessed in a supportive listening approach. However, our pilot work and our use of ISP in other trials suggest this possibility is limited. Further, our fidelity coding plan includes assessing each session for any such contamination. It is also notable that the target patient group is a relatively small patient population. However, preliminary testing of the recruitment plan and a clinical data review suggest the likelihood of success in obtaining the sample. Also, a portion of the success of the study will rely on proper sample collection at home. Use of online and smart technologies may be helpful in this regard. A demonstration video of proper saliva collection and sample handling will be made available to all participates. Likewise, new smartphone applications have been designed to specifically record the timing and compliance of at-home sampling. Finally, it should also be acknowledged that the distress screening measure is different from the depression and anxiety outcome measure. This strategy proved effective in pilot work. The use of the DT as a screening tool allowed for potential participants to identify diffuse experiences of distress, rather than endorsement of specific depressive symptoms.

Dependent on our findings, future directions will include a larger efficacy trial, with a focus on examining impact on longer-term (and late) adverse effects by including a longer-term follow-up period, recognizing the potential future physical and psychological vulnerabilities of this population. An additional future priority will be to test the intervention across cancer types. It will also be important to identify differential effects of each intervention component, and determination of who responds best to each component for better optimization. Finally, future studies will also evaluate the feasibility, acceptability, and efficacy of the delivery of GET via mobile application or telehealth modalities.

Findings from this study will be reported in line with CONSORT standards for reporting pilot and feasibility trials [65, 66], and will inform the possibility of a future larger-scale trial which has potential to impact long-term cancer survivorship in a population largely underserved by typical cancer survivorship care. Aligned with recommended goals of pilot studies [67], the primary conclusions of this study will be related to decisions on whether to proceed and how to proceed with a future efficacy trial. Observations of change will be treated with caution and considered preliminary. Study results as they pertain to the aims of this pilot study will be communicated to relevant scientific and clinical communities via peer-reviewed publications, scientific and clinical conference presentations, and report writing for lay audiences. In addition, if our biobehavioral approach is supported, it will inform the biological pathways and risk factors that influence the negative adverse impacts of cancer and cancer treatment over the long term.

## Trial status

This trial was initiated in January 2017. Recruitment is ongoing and expected to complete by October 2020. This protocol is version 2.

## Data Availability

Not applicable.

## Abbreviations

ANCOVA: Analysis of covariance
CAYA-T: Cancer Assessment for Young Adults—Testicular
CTI: Career Thoughts Inventory
CRP: C-reactive protein
DT: Distress Thermometer
EDTA: Ethylenediaminetetraacetic acid
ERQ: Emotion Regulation Questionnaire
GET: Goal-Focused Emotion-Regulation Therapy
HADS: Hospital Anxiety and Depression Scale
HPA: Hypothalamic–pituitary–adrenal
IL-1RA: Interleuken-1 receptor agonist
IL-6: Interleuken-6
ISP: Individual Supportive Psychotherapy
sAA: Salivary α-amylase
SAM: Sympathetic adrenal medullary
sTNFr: Soluble tumor necrosis factor receptor 2
VEGF: Vascular endothelial growth factor
